# Assessing a pilot co-operative-based workshop-subsidy model toward improving small-scale chicken production in peri-urban Nepal

**DOI:** 10.1093/tas/txac071

**Published:** 2022-06-30

**Authors:** Sarai Acosta, Ruby Chen, Sunita Nhemaphuki, Damber Khanal, Myrna Cadena, Maurice Pitesky, Nancy Erbstein, Deb Niemeier

**Affiliations:** Department of Population Health and Reproduction, School of Veterinary Medicine, University of California, Davis, CA 95616, USA; Department of Civil and Environmental Engineering, University of Maryland, MD 20742, USA; Civil and Environmental Engineering, UC Davis College of Engineering, 1 Shields Ave. Davis, CA 95616, USA; R&D Innovative Solutions, Kathmandu, Nepal; R&D Innovative Solutions, Kathmandu, Nepal; Department of Population Health and Reproduction, School of Veterinary Medicine, University of California, Davis, CA 95616, USA; Department of Population Health and Reproduction, School of Veterinary Medicine, University of California, Davis, CA 95616, USA; School of Education, University of California, Davis, CA 95616, USA; Department of Civil and Environmental Engineering, University of Maryland, MD 20742, USA

**Keywords:** coop design, Nepal, small-scale poultry, work-shop subsidy model

## Abstract

Farmers in Nepal face many of the same global challenges associated with initiating and scaling poultry husbandry as many other developing countries. These include access to innovative approaches in finance, credit, coop design, marketing, and sales. As with most low-income countries, Nepalese poultry farmers also lack adequate training in poultry husbandry including biosecurity. In this paper, we describe a collaborative workshop-subsidy approach to addressing these challenges conducted by a partnership with the UC Davis School of Veterinary Medicine, the College of Engineering, the School of Education, and a farming co-operative based in the semi-rural area of Bhaktapur, Nepal. The program included two workshops covering aspects of poultry rearing including coop construction, chick rearing, biosecurity, and husbandry. Both workshops were a combination of lectures and hands-on learning. Following completion of the workshops, each farmer received subsidized materials for coop construction and poultry rearing. The co-operative provided training facilities and a market for selling eggs. Despite an outbreak of Highly Pathogenic Avian Influenza (HPAI), which affected the scale of program implementation, our results suggest that the workshop subsidy collaborative approach can be successful in reducing market entry barriers. Our 6-mo post-workshop survey showed that two-thirds of the workshop participants ultimately built their own coop and raised chicks. Half of these participants reported market available egg production and a doubling of egg consumption at home.

## INTRODUCTION

Village poultry and small-scale chicken production that may or may not use village (i.e., indigenous) breeds play a significant role in food security, poverty alleviation, and rural development in the developing world (Permin et al., 2001; Mack et al., 2005; Alders and Pym, 2009; Schmidt, 2017). The benefits of village poultry include low capital and operating costs, supplying eggs and meat for home consumption and commercial sale, facilitating pest control and providing a rich source of fertilizer for crop production (Mack et al., 2005; Alders and Pym, 2009). The challenges associated with village poultry relative to conventional production can include poor production, high levels of infectious disease (e.g., virulent Newcastle disease [vND] and Avian Influenza [AI]) and increased mortality due to predation (Thekisoe et al., 2004; Dinka et al., 2010). Although small-scale poultry may be less profitable than larger conventional commercial production systems, the low initial capital and operating costs make it practical for individual households to consider in much of Africa, Asia, and Latin America (Bell, 2009; Alders et al., 2018).

Efforts to increase village/small-scale production efficiencies traditionally center on disease prevention with a focus on vaccination against diseases with high morbidity and mortality including vND and HPAI (Wondmeneh et al., 2016). Although additional relevant topics such as coop design and general business operations are also covered in the literature (Alders, 2004; Sonaiya and Swan, 2007), comprehensive programs/studies that integrate multiple relevant factors (e.g., training, marketing, finance, and coop design and construction) for small-scale production are less common. One of the more comprehensive approaches to training is the “Bangladesh Model,” a semi-scavenging poultry approach with a focus on empowering women (Dolberg, 2001; Permin et al., 2001). The main model constituents include NGOs that provide training and micro-credit for poor women farmers (Dolberg, 2001).

Our work expands on the basic “Bangladesh Model” for commercial village poultry production through the use of a co-operative-based workshop-subsidy format to introduce coop design, construction, and commercial intent. Similar to NGOs, co-operatives are commonly found in many countries and across many sectors including agriculture (Kumar et al., 2015; Schmidt, 2017). Unlike NGOs, co-operatives are typically local for-profit entities made up of participants with common economic (among other) goals (Kumar et al., 2015). Because most farms in the developing world are small/subsistence farms, co-operatives offer an organizational form that has the potential to integrate and expand economic growth and opportunity (Kumar et al., 2015) for commercial village poultry producers (IFAD, 2021). Although the evidence of co-operative success is mixed (Kumar et al., 2015; Shah, 2020), research is consistent in finding that co-operatives formed at the community level tend to outperform those established through the government or an outside NGO (Sarker et al., 2016; Zou et al., 2016). This is an important observation for many countries that do not have strong central governments in the sense that lack of a strong central government may not be a significant impediment toward expansion of small/subsistence commercial farming.

**Figure 1.**
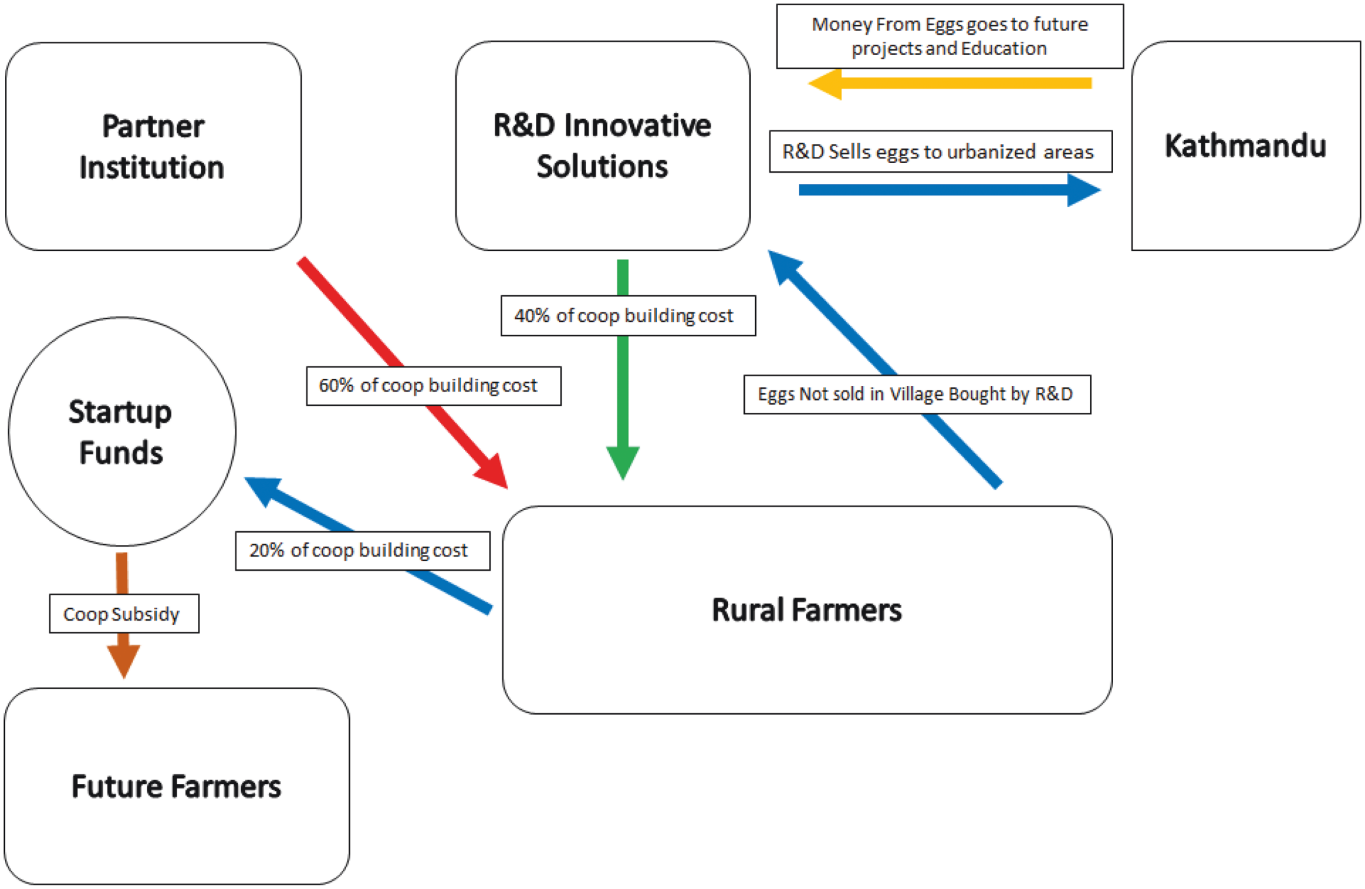
Economic flow chart showing the subsidy provided by UC Davis and R&D Innovative Solutions to the 16 farmers who are part of the R&D collaborative.

In this project, UC Davis and a Nepal-based co-operative, R&D Innovative Solutions (referred to as R&D for the remainder of the manuscript) in the Bhaktapur region, teamed to pilot a novel workshop-subsidy format for new poultry farmers (Figure 1). R&D is an established co-operative that collects and directly distributes organically grown products to the larger Kathmandu area. R&D took leadership in recruiting motivated commercial (primarily female) farmers within their co-operative to participate in the program, which consisted of two workshops. The first workshop delivered training in the basics of poultry husbandry. The second, more intensive workshop focused on coop construction, biosecurity, and chick rearing. Farmers completing both workshops were provided subsidized equipment and supplies to build coops and raise chicks for egg production. Under this model, farmers sell eggs to the R&D co-operative at a pre-arranged price (Figure 1). In this paper, we present the results of a follow-up evaluation conducted approximately 8 mo after the second workshop.

## MATERIALS AND METHODS

A workshop-subsidy model was developed in collaboration with R&D as described in Figure 1. Multiple meetings between the cooperative (R&D) and UC Davis over time were used to develop both the work-shop subsidy model and the didactic and hands on training, in order to ensure that they reflected the interests, aspirations, knowledge needs, and learning styles of participants. In short, the model created a mechanism to facilitate training and acquisition of operating and capital supplies to support commercial village poultry for co-operative farming members. The program targeted female R&D co-operative member farmers interested in expanding their commercial farming operations to include commercial village layer poultry. Participants were required to complete both workshops to be eligible for a subsidy of approximately 80% of the costs associated with coop construction supplies; chicks and chick starter feed acquisition were provided. The subsidized costs were divided 60% and 40% between UC Davis and R&D, respectively. R&D purchased eggs from their co-operative members that were sold in the larger Kathmandu area. Twenty percent of sales were set aside as a “start-up” fund to provide subsidies for future farmers (Figure 1).

### Workshops and Recruitment of Participants

The workshops were held in the city of Gundu in December, 2018 and March, 2019, the first (two-day) workshop was primarily didactic covering care for laying hens, coop design, disease prevention, poultry nutrition, and food safety. The second (three-day) workshop was primarily interactive, with a focus on chick rearing (i.e., brooding, vaccination against Marek’s disease and vND, and basic chick management) and coop construction. With workshop participants helping, construction of two of the mobile coops during the second workshop was accomplished. An instructional booklet on how to build the coop written in Nepali was developed and handed out to each participant in attendance.

Presentations were given in English with Nepali translation. Translation of didactic material was done in collaboration with R&D and a Nepalese visiting student at UC Davis. Workshop times, locations, and agendas were carefully designed with local input to facilitate participant engagement. Participation in the second workshop was contingent on participation in the first workshop or via permission of R&D. Sixteen co-operative farmers participated in the second workshop.

### Coop Construction

**Figure 2.**
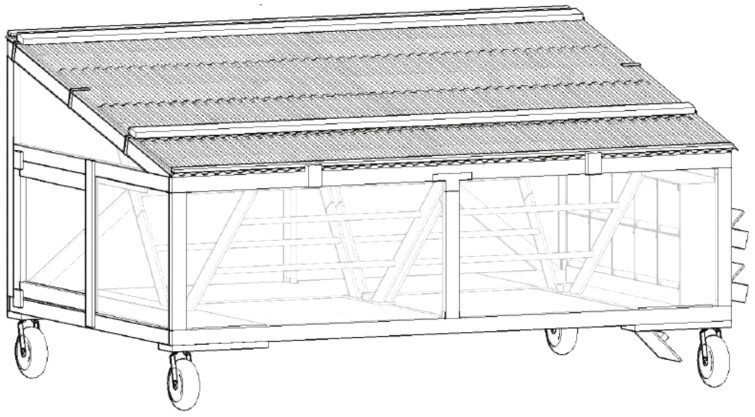
Drawing of the Eggmobile designed by Ruby Chen and Deb Niemeier from the UC Davis Sustainable Design Academy. The coops were originally designed and built for the UC Davis Pastured Poultry Farm. This design was then used as part of the second workshop in Nepal where two demonstration coops were built by the 16 co-operative farmers. Following the completion of the workshop, all 16 farmers-built coops back at their homes with materials costs subsidized by R&D and UC Davis.

A large component of this project focused on developing a local resourced new village poultry mobile coop design that was flexible in fabrication, easy to construct, and scalable. Students participating in the UC Davis Civil Engineering senior design course (ECI 193A) developed a new, mobile coop design in 2018 (Farm 2018). The coops are approximately 366 × 244 cm (length × height) with a tapered roof that spans 110 to 145cm (Figure 2). The interior provides for 10 nest boxes, and roosting bars. Assuming at least 139cm^2^ of space per bird and 1 nest box per 5 birds, the coop capacity was calculated to be approximately 50 layers or up to 64 broilers. The design allows for locally sourced materials to be utilized. In Nepal, the frame was constructed of bamboo and wire mesh in the main structure and corrugated galvanized iron (CGI) for the roof. Supply costs in NPR and USD are provided in Table 1 using prices at the time of purchase, March 2019.

**Table 1.** Coop design characteristics for one coop

Coop construction supplies and cost					
Material	Unit	Cost per unit (NPR)	Unit	Subtotal cost (NPR)	Subtotal cost (USD)
Bamboo	20' Piece	240	16	3,840	34.94
Wire Mesh	4' Wide, Feet	80	50	4,000	36.40
GI Wire	Kg	150	3	450	4.10
Tarpaulin Sheet	Sq. Ft.	6.5	254	1,651	15.02
CGI	6' Long Sheet	467	6	2,800	25.48
Plastic Crate	General Sized Crate	400	3	1,200	10.92
	1USD = 0.0091 NPR NPR = Nepalese Rupee		Total Cost (TC)	13,941	126.86
			60% of TC	8,365	76.12
			40% of TC	5,576	50.75

The cost per unit was 13,941 NPR or $126.78 USD.

## RESULTS

In the 4 wk following the second training, 16 additional chicken coops were constructed at the homes of workshop participants in collaboration with the training staff. Once the new coops were completed, ten of the 16 participants (62.5%) received 30 to 45 baby chicks for rearing from a local hatchery. Six of those completing construction of the coop did not receive chicks due to a local outbreak of HPAI, which resulted in a regional quarantine. Following coop construction and placement of chicks a post-workshop evaluation using a structured interview with the assistance of a translator was administered approximately 8 mo after the second workshop in November of 2019. The survey compromised two parts: a demographic survey (Table 2) and a production survey (Table 3). The survey was determined to be exempt from Institutional Review Board (IRB) approval by UC Davis.

**Table 2.** Demographic and egg usage data collected from the 10 farmers who built coops and were provided with chicks

ID	Primarycaretaker	HHsize	Men in HH (N)	Women in HH (N)	Hens laying	Rent land	Own land	Eggs gifted	Eggs sold	Eggs bought	Eggs for brooding	Increased HH egg consump
1	male	6	3	3	•	•			•		•	•
2	female	5	2	3		•		•		•		
3	male	5	2	3		•				•		
4	female	3	2	1	•	•			•		•	•
5	female	3	2	1			•			•		
6	NA	NA	NA		NA	NA	NA	NA	NA	NA	NA	NA
7	female	6	3	3	•		•		•	•	•	•
8	female	4	3	1	•		•				•	•
9	male	6	3	3	•		•	•	•		•	
10	female	6	4	2			•					

Surveys were completed when hens were approximately 38 wk of age.

**Table 3.** Production data collected from the 10 farmers who built coops and were provided with chicks

Table of poultry data findings	
Egg production data from the 5/10 participants who had laying hens	
Average number of eggs eaten weekly	20.70
Average number of eggs laid per week	28.80
Range of eggs lain a week	25–45
Biosecurity and welfare adoption	
Number of farmers who had roosting bars in coop	6
Average time hens spent on roosting bars	2–3 h
Number of coops with nesting boxes	6
Number of next boxes used	6
Were waterers cleaned daily	10
Were feeders cleaned daily	10
Number of participants who let their birds outdoors	9
Average time birds spent outdoors	2–3 h/d
Feeding	
Were birds fed daily	10
Feed type(s) used	
Chicken feed	9
Grains	9
Maize	4
Table scraps	5
Vegetables	6
Wheat	4
Rice	3
Morbidity and mortality	
Farmers reporting poultry mortality	1
Farmers reporting morbidity	5
Farmers reporting animal attacks	3

Surveys were completed when hens were approximately 38 wk of age.

### Demographic and Egg Usage Information

Of those receiving chicks, six out of the 10 post-workshop participants (60%) reported women as the primary caretaker (Table 2). The six female caretakers came from households ranging from 3 to 6 persons, whereas the three male caretakers (one household did not provide a response) came from larger households (5 + persons). Female caretakers were also more likely to own their land (four of six) than rent (Table 2). Of those farmers reporting that they had sold no eggs (five), three out of five households were still buying eggs (Table 2). Of those reporting that they were selling eggs, three out of four also reported that they were not buying eggs. Three of the four households reporting eggs being sold also indicated eggs were being consumed (Table 2).

### Layer Production and Human Egg Consumption Results

Farmers reported an average of 28 eggs consumed per week (Table 3). With a reported average of about 29 eggs per week reported as laid, only a small portion of the eggs were going to market in weeks with low numbers of eggs. Farmers also reported a loss of eight to nine eggs per week. Mortality was relatively low with only one of the ten farmers reporting any mortality (Table 3). Most farmers reported feeding hens mostly chicken feed and grains followed by other feeds including table scraps and vegetables.

### Basic Husbandry

Five of the 10 farmers who had chicks reported egg production after 28 wk. Farmers reported daily cleaning of watering and feeders as well as daytime scavenging. With respect to roosting bars the uptake rate for inclusion of nest boxes and roosting bars was 40% (6/16) (Table 3).

### Coop Utilization and Biosecurity

Based on discussions with R&D, the original design was aimed at a price point of approximately $150 USD. The final design came in at $126 USD. The highest material cost in both the Nepal version and the U.S. pilot coops was the wire mesh, with bamboo (Nepal) and wood (United States) following behind. In both cases, labor costs were not integrated into the cost calculation. With respect to building coops for each individual farmer, farmers were encouraged to decide for themselves what aspects of the coop design were necessary. To that point, the post-workshop evaluation showed that 6 of the 10 farmers built and used the nest boxes (Table 3).

## DISCUSSION

Although village poultry is a fundamental aspect of food security in the developing world (Wong et al., 2017), multiple inefficiencies exist at the economic and production level (Nduthu, 2015). Approaches toward addressing these inefficiencies in a practical manner focus largely on disease prevention (Thekisoe et al., 2004) and production improvements (Copland and Alders, 2005). However, the ability to leverage the infrastructural advantages of co-operative systems (Novkovic, 2008) including access to commercial markets, motivated farmers and access to subsidized training, operating, and capital costs to support expansion of farming enterprises have not been well studied for village poultry. Here a novel workshop-subsidy model for new poultry farmers associated with an existing agricultural co-operative was developed (Figure 1) in order to leverage the co-operatives existing market (Figure 2). Results demonstrated a high level of participation at the farmer level. Specifically, of the 16 R&D co-operative farmers that participated in the second workshop, all of them built coops, ten of the 16 participants in the training received 30 to 45 baby chicks for rearing from a local hatchery and 5/16 reported either selling or gifting eggs (Table 3). The remainder did not have chicks delivered due to a regional outbreak of HPAI that resulted in a quarantine.

It could be hypothesized that improvements in village poultry (e.g., participation, production, education, and profitability) could be partially realized via this approach. Although this project was not structured to answer this question, at a pilot-cooperative level, it did demonstrate the potential for the workshop-subsidy approach toward the expansion of local egg production for small farmers for both home consumption and for sales within a cooperative system. Although the majority of the table eggs were consumed by the farmers themselves (Table 3) as opposed to being sold through the co-operative, one could argue that the farmers decision making likely prioritized home consumption as opposed to sales and that this would change with even a slight increase in production. Likely the potential for further expansion to support additional sales will depend on multiple factors linked to economics and food security at the farmer level. However, with the hands on training and skills gained in husbandry, coop design, and building, future expansion within the co-operative is potentially more likely since co-operatives have been found to be more likely to adopt various innovations (Abnousi et al., 2020). This improved access to innovation is likely due to access to social capital defined as the ability to develop and use various social networks and the resources associated with them (Parthasarathy and Chopde, 2000).

However, even with the social capital associated with membership in the R&D co-operative, without the provided 80% subsidy (Figure 1), the current mobile coop’s cost of approximately $126 (Table 1) is a significant capital cost to village poultry owners in rural Nepal where the median salary is approximately $8400 (Explorer, 2021). Because village poultry have consistently been reported to be marginally profitable (Francis et al., 2016; Wondmeneh et al., 2016), the question of whether production and profitability could be improved using this workshop subsidy-model is a fundamental question toward addressing the challenges of village poultry production.

However, from a development perspective, economics is only one factor in the overall value of village poultry. For example, village poultry are often owned by women and the landless (Guèye 2000, Mack et al., 2005). Six of the ten farmers who reared chicks were female and five of the ten farmers identified themselves as owning their land. To that point, there is strong global evidence that demonstrates the relationship between female village poultry ownership and improved women’s education and empowerment (Sodjinou, 2011; Kumar et al., 2015). With respect to land ownership, there is a significant body of literature that suggests that land ownership is a significant factor in farmer profitability (Alam et al., 2010; Hofstrand, 2015). However, there is also a significant amount of literature that suggests that access to credit especially for small farmers in the developing world is a significant constraint on agricultural expansion and productivity (Foster and Rosenzweig, 2010). Consequently, the described farmer-subsidy model may have some positive effect on village poultry with respect to expansion for established low-income farmers keen to try village poultry with minimal risk.

Additionally, highly infectious poultry diseases such as vND and HPAI are relatively common in Asia and Africa which is in part due to the high prevalence of village poultry and the general lack of biosecurity commonly associated with village and small-scale chicken production (Awan et al. 1994, Mack et al., 2005). Modes of disease transmission within and between villages including fecal-oral, respiratory, and contact with wild birds are likely exacerbated by this lack of biosecurity associated with village poultry production (Awan et al., 1994). Consequently, the utilization of coops could play an important role in both biosecurity and production efficiency which has been shown to have a positive economic impact (Otte et al., 2021). However, as soon as chickens are confined the ability to provide feed (as chickens are not able to scavenge) becomes relevant and is not common (Otte et al., 2021) Interestingly, the ultimate value of a coop-based system in the village poultry environment has not been studied with respect to biosecurity, production, and economics likely due to the reality that most village poultry are historically free-ranging/scavenging (Alders and Pym, 2009). One well-described example that utilizes coops is the Helen Keller International model that utilized a similar approach with respect to training and inputs with a limited (15%) uptake of “best practices.” (Nordhagen and Klemm, 2018). In our case, HPAI occurred in commercial poultry and resulted in a regional quarantine which prevented further placement of chicks. This demonstrates the reality that infectious diseases can spread to all poultry (village and conventional) and result in quarantine and other consequences (e.g., trade embargo and depopulation) for unaffected poultry in different production systems poultry (Alders et al., 2014).

With respect to productivity, our results were largely consistent with prior literature. For example, with respect to productivity, the range of chicks provided to each farmer (30–45) vs. the average egg production per week (20.8) (Table 3) reflected low productivity likely due to morbidity, mortality, and poor nutrition and husbandry (Table 3) which have also been noted in other studies and reviews of village poultry production (Kingori et al., 2010; Kingori et al., 2010; Nduthu, 2015). Although this study was not set up to identify any differences in production between village poultry with “innovations” such as roosting bars and egg coops, there appeared to be no difference between farmers that used/did not use those “innovations.” Hence, although the potential advantages of having nest boxes are that the majority of eggs are laid in the nest boxes making eggs cleaner, less likely to break and easier to collect and the potential advantages of having roosting bars include improved welfare (Duncan et al., 1992) and increased bone density (Hester et al., 2013) which could indirectly increase egg production, no such difference was observed. Further cost/benefit analyses of these “innovations” in village poultry are necessary to better understand the value of nest boxes and perches in a village system.

In addition to the coop, an additional expense integrated into the workshop-subsidy model was access to the commercial feed for the chicks. The cost-benefit of commercial feed vs. scavenging, the most common source of calories for village poultry, is poorly understood. We attempted a hybrid approach where 2 bags of chick starter feed were provided to each co-operative farmer upon receiving their chicks with the logic that the highest mortality usually occurs as chicks and that proper nutrition would reduce mortality and increase productivity. Our experimental design and survey structure were not designed to evaluate the efficacy of this approach but further investigation of this type of approach is likely warranted.

Although our post-workshop evaluation suggests that eggs are primarily used for home consumption (Table 3) as opposed to commercial sales, the ability to raise laying hens for home egg production (and eventually meat) is considered an important aspect of poverty alleviation (Permin et al., 2001; Jensen and Dolberg, 2003). Based on the number of eggs consumed, the range of possible egg production, and the size of the families, a minimum of over 28 eggs a week would be necessary to accommodate additional egg sales (Table 3). Further study is required to determine the effects of home consumption on farmer income.

## CONCLUSION

The workshop-subsidy co-operative model described here, between a University and a local agricultural co-operative, demonstrates a unique approach for improving village poultry production. However, the ultimate value and sustainability of this approach is debatable and ultimately depends on which metrics (economics, food security, malnutrition, and women empowerment) define success. The objective of reducing poverty and malnutrition is multi-factorial in complexity and scope and no single intervention will likely have a major impact (Mack et al., 2005). However, given this reality, even modest improvements in the economics, food security, nutrition, and women’s empowerment are important. If this approach is deemed to be successful, universities/NGOs should look to partner with local co-operatives that are keen to expand their production. This approach has the potential to amplify the overall impact by partnering with co-ops as opposed to recruiting individual farmers.

To this point, delivery of our workshops resulted in improvements in village poultry production and improved biosecurity practices. The overall uptake of the coops was 100% and the uptake of farmers with coops with chicks was 62.5% (10/16), even with the constraints of an HPAI outbreak, which indicates a reasonably successful pilot offering. Although it is likely that based on previous data these uptake rates will drop (Nordhagen and Klemm, 2018), continued efforts such as our pilot may have a greater effect as other external infrastructural factors change in-country including access to vaccination, feed, healthy chicks, and allied supplies (Nordhagen and Klemm, 2018).
